# Effects of creatine supplementation on oxidative stress profile of athletes

**DOI:** 10.1186/1550-2783-9-56

**Published:** 2012-12-21

**Authors:** Sandro Percário, Sérgio Paulo de Tarso Domingues, Luiz Felipe Milano Teixeira, Jose Luiz Fernandes Vieira, Flavio de Vasconcelos, Daiane Marques Ciarrocchi, Eduardo Dias Almeida, Marcelo Conte

**Affiliations:** 1 Biological Sciences Institute, Federal University of Pará, Belém, PA, Brazil; 2 Anhanguera College, Sorocaba, SP, Brazil; 3 Jundiaí Physical Education College, Jundiaí, SP, Brazil; 4 Health Sciences Institute, Federal University of Pará, Belém, PA, Brazil; 5 Universitary Centre FIEO, Osasco, SP, Brazil

**Keywords:** Free radicals, Antioxidant capacity, Resistive training, Uric acid, Thiobarbituric acid, Oxidative stress, Athletes

## Abstract

**Background:**

Creatine (Cr) supplementation has been widely used among athletes and physically active individuals. Secondary to its performance-enhancing ability, an increase in oxidative stress may occur, thus prompting concern about its use. The purpose of this study is to investigate the effects of Cr monohydrate supplementation and resistance training on muscle strength and oxidative stress profile in healthy athletes.

**Methods:**

A randomized, double-blind, placebo-controlled method was used to assess twenty-six male elite Brazilian handball players divided into 3 groups: Cr monohydrate supplemented group (GC, N = 9), placebo group (GP, N = 9), no treatment group (COT, N = 8) for 32 days. All subjects underwent a resistance training program. Blood samples were drawn on 0 and 32 days post Cr supplementation to analyze the oxidative stress markers, thiobarbituric acid reactive species (TBARS), total antioxidant status (TAS), and uric acid. Creatine phosphokinase, urea, and creatinine were also analyzed, as well. Fitness tests (1 repetition maximum - 1RM and muscle endurance) were performed on the bench press. Body weight and height, body fat percentage (by measuring skin folds) and upper muscular area were also evaluated. Statistical analysis was performed using ANOVA.

**Results:**

Only GC group showed increase in 1RM (54 ± 9 vs. 63 ± 10 kg; *p* = 0.0356) and uric acid (4.6 ± 1.0 vs. 7.4 ± 1.6 mg/dl; *p* = 0.025), with a decrease in TAS (1.11 ± 0.34 vs. 0.60 ± 0.19 mmol/l; *p* = 0.001). No differences (pre- vs. post-training) in TBARS, creatine phosphokinase, urea, creatinine, body weight and height, body fat percentage, or upper muscular area were observed in any group. When compared to COT, GC group showed greater decrease in TAS (−0.51 ± 0.36 vs. -0.02 ± 0.50 mmol/l; *p* = 0.0268), higher increase in 1RM (8.30 ± 2.26 vs. 5.29 ± 2.36 kg; *p* = 0.0209) and uric acid (2.77 ± 1.70 vs. 1.00 ± 1.03 mg/dl; *p* = 0.0276).

**Conclusion:**

We conclude that Cr monohydrate supplementation associated with a specific resistance program promoted a meaningful increase in muscle strength without inducing changes in body composition. The observed significant increase in uric acid and the decrease in TAS suggest that creatine supplementation, despite promoting acute effects on muscle strength improvement, might induce oxidative stress and decreases total antioxidant status of subjects.

## Background

Creatine (Cr) supplementation has been widely used among athletes and physically active individuals. Since the beginning of the 1990s, the estimated Cr consumption in the United States alone has reached approximately 2.5 million kg/year [1], and has been one of the most studied ergogenic resources in recent years [2]. In the last 20 years, many authors have suggested that Cr supplementation may be an effective ergogenic aid for exercise and sports [3].

Although clinical studies of Cr supplementation have speculated the occurrence of side effects [4], extensive literature reviews conducted by the American College of Sports and Medicine [1], and more recently by the International Society of Sports Nutrition [5], concluded that such complications were not actually observed in the analyzed studies and reached a consensus that Cr supplementation is a safe practice when administered within the recommended criteria.

Since the 1980s, accumulating evidence indicates that strenuous exercise or unsystematic physical activity entails an imbalance between free radicals and the antioxidant defense system by significantly rising free radical production, and drastically reducing total antioxidant capacity, leading to oxidative stress as inevitable consequence [6,7]. Although oxidative stress can manifest itself in different ways, during exercise it often leads to lipid peroxidation, caused mainly by the action of hydroxyl radicals (OH^·^) on unsaturated lipids [8-11], and to the occurrence of ischemia-reperfusion syndrome.

Indeed, ischemia-reperfusion syndrome is one of the most important problems indentified in the production of free radicals. Resistance training is believed to induce ischemia-reperfusion injury owing to the fact that it combines static and dynamic muscle contraction during the resistance training proportional to the effort required to move the weight. This mechanism promotes a number of important hemodynamic responses, for example, increased systolic and diastolic blood pressure and heart rate with concomitant relative increase in peripheral resistance to blood flow [12]. Since resistance exercises consist of short term and high intensity sessions, their primary energy source is the anaerobic production of ATP. During short-duration, high-intensity exercise, the anaerobic pathways of ATP resynthesis are not always sufficient to meet the energy demands. Therefore, the hydrolysis of ADP to AMP is required, leading to the final hypoxanthine formation.

However, a substantial reperfusion occurs in muscles during the intermediary process, thus creating the appropriate environment for free radical formation from ischemia-reperfusion syndrome [13].

Few studies have been published concerning the relationship between Cr supplementation and free radical-induced oxidative stress. Nevertheless, reported results are controversial and inconclusive. Accordingly, resistance-trained men underwent a 7-day Cr supplementation (20g/day Cr monohydrate) or placebo (PL) supplementation. During supplementation the subjects performed a resistance exercise protocol. Plasma malondialdehyde (MDA) and urinary 8-hydroxy-2-deoxyguanosine (8-OHdG) were measured. Cr supplementation caused a significant increase in athletic performance attenuating the changes observed in the urinary 8-OHdG excretion and plasma MDA, suggesting that Cr supplementation reduced oxidative DNA damage and lipid peroxidation associated to resistance training [14].

On the other hand, adult males performed repeated exhaustive incremental cycling trials and received Cr or placebo supplementation. Breath-by-breath respiratory data and heart rate were continually recorded throughout the exercise protocol; blood samples were drawn at resting state 20 minutes after stopping exercises and on the day following the exercise. The results showed that supplementation did not influence lipid peroxidation, resistance of low density lipoprotein to oxidative stress or plasma concentrations of non-enzymatic antioxidants. Heart rate and oxygen uptake responses to exercise were not affected by supplementation, whereby the authors concluded that short-term creatine supplementation does not enhance non-enzymatic antioxidant defenses or protect against lipid peroxidation induced by exhaustive exercise [15].

However, findings about the correlation between Cr supplementation and free radical production could help to understand if increase in intramuscular creatine can reduce the formation of hypoxanthine and prevent ischemia reperfusion syndrome; or if great Cr availability could increase oxidative stress, since more energy is generated. Therefore, the purpose of this study is to verify the effects of Cr supplementation and intense resistance training on muscle strength and oxidative stress of athletes.

## Methods

### Subjects

Twenty-six male handball athletes (17.10 ± 1.63 years; ranging from 15 to 19 years old) from Sorocaba, SP, Brazil participated in this experiment. Exclusion criteria were: i) no previous experience in resistance training, ii) current use of any nutritional supplement, iii) current or previous intake of anabolic androgenic steroids, iv) current or previous intake of Cr and maltodextrin for supplemental purposes, and v) pre-existing abnormalities revealed in laboratory tests or medical exam at the beginning of experimental analysis. All experimental procedures were performed in accordance with the Helsinki Declaration and the guidelines established by the Brazilian National Committee of Research on Human Subjects. The Catholic University Human Subjects Ethics Committee approved all experimental procedures. All subjects provided written consent prior to participating in this study according to the Brazilian Ministry of Health/National Health Foundation.

### Experimental procedures

A randomized, double-blind, placebo-controlled study with subjects divided into 3 groups: GC (N = 9) Cr monohydrate supplemented, GP (N = 9), a placebo group that consumed maltodextrin [16], and COT (N = 8), a group of athletes who did not receive Cr or placebo. All individuals (GC, GP, and COT) underwent a 32-day resistance training program that began and finished concomitantly with Cr and Placebo supplementation. One day prior to Cr/Placebo supplementation and resistance training, and one day following completion of Cr/Placebo supplementation and resistance training, a blood sample from the cubital vein was drawn for verification of oxidative stress parameters, body composition was assessed, and muscle strength and endurance tests were applied to all athletes. All athletes were examined by a sports medicine doctor before, during, and after completion of study. No abnormalities were found in subjects’ health condition at any time during the survey. The protocol used for clinical examination was carried out in accordance with the recommendations from the International Olympic Committee. Moreover, subjects were asked weekly about the occurrence of the following symptoms: increased thirst, fatigue, frequent headaches, frequent irritability, tinnitus, numbness in the head, neck, back, or limbs, shivering and chills, nausea, diarrhea, stomach discomfort, cramps, and dizziness. All athletes had frequent consultations with the team’s physician about the occurrence of muscle or joint injuries or other clinical conditions.

### Supplementation protocol

During the first 5 days of the protocol, Cr monohydrate was administered at a daily dose of 20 g, taken as 4 doses of 5 g each, dissolved in 100 ml of water [17]. On the remaining 27 days, participants were given a dose of 5 g Cr per day, diluted in 100 ml of water, after training. All doses were taken before a member of the researchers’ crew. Creatine supplements were obtained from a local supplier (Integral Medica; São Paulo-Brazil). Placebo was administered with the same protocol to GP athletes, and contained only maltodextrin. The dosage regimen was established according to observations from other studies, in which variations between 4 and 12 weeks of supplementation were employed [1,2,16,18,19]. Additionally, during the study period, all participants were instructed not to modify their usual diets; all dietary information of athletes, who lived in research facilities and had breakfast, lunch and dinner prepared by same cook, was recorded throughout the study.

### Resistance training protocol

All volunteers underwent the same specific training program of periodized resistance (Table 1) concurrently with the initial administration of Cr supplementation. The training was conducted in 4 phases: familiarization, hypertrophy, strength, and peak. The objective was to increase maximum force using a classical linear periodization protocol [19,20]. The athletes had previous experience on resistance training. Unless participating in the regular physical training with the team, they were instructed not to perform any activity or physical training other than the exercises carried out in the present study so as to avoid interference in the response to training. The exercise intensity for the resistance training program was determined according to the principle of 1-repetition maximum (1-RM), as described by the American College of Sports and Medicine [21]. The RT sessions were identical with regard to the sequence and exercises used during periodization: 1) Bench press; 2) Inclined Chest Fly; 3) Lat pull down; 4) Seated Row; 5) Shoulder press 6) Biceps curl; 7) Squatting; 8) Leg Extension.

**Table 1:** Characteristics of the resistance training periodization

**VARIABLES**	**METHOD**			
	**Familiarization**	**Hypertrophy**	**Strength**	**Peak**
Duration	1 week	2 weeks	1 week	1 week
Intensity	50% 1RM	75–80% 1RM	80–85% 1RM	85–95% 1RM
Repetitions	12	8–10	6–8	3–6
Sets	3	3	4	3
Interval between sets	90 s	90 s	120 s	180 s
Speed of repetitions	Moderate	Moderate	Moderate	Moderate
Frequency	3 times/week	4 times/week	3 times/week	3 times/week
Exercises per session	8	8	7	7

Moderate speed: one second in concentric phase and two seconds in eccentric phase.

### Blood collection

At the beginning and end of the supplementation period, blood samples were collected from volunteers by cubital vein puncture and placed in vacuum test tubes containing sodium heparin. Plasma was obtained by centrifugation at 2500 rpm for 15 min.

### Laboratory testing

Routine biochemical testing was performed; creatine phosphokinase (CPK), creatinine, and urea were evaluated spectrophotometrically using commercial kits (Labtest Ltda; São Paulo-Brazil). The occurrence of oxidative stress was assessed through the plasma levels of thiobarbituric acid reactive substances (TBARS), a widely used indicator of lipid peroxidation; uric acid, a byproduct of ischemia-reperfusion syndrome, and total antioxidant status (TAS), as well. Uric acid was assayed using commercial kits (Labtest Ltda; São Paulo-Brazil) in a UV/VIS photometer (Fento Ltda.; São Paulo-Brazil). TBARS determination was performed by the Khon & Liversedge method (1944), modified by Percario et al. [22], in which 0.5 ml of plasma was added to 1.0 mL of thiobarbituric acid reagent (10% in PBS solution; pH=7.2), heated at 95°C for 60 min, extracted with 4.0 mL of butylic acid, and centrifuged at 3000 rpm for 15 min. Supernatant was then collected and spectrophotometrically measured at 535 nm (Fento Ltda.; São Paulo-Brazil). TAS was assayed according to the method described by Re et al. (1993) [23] using the Total Antioxidant Status Kit (Randox Laboratories Ltd., NX2332). Briefly, 20 μL of sample is added to 1.0 mL of ABTS® reagent and the absorbance reduction rate at 600 nm was recorded (Fento Ltda.; São Paulo-Brazil). For TBARS 1,1,3,3 tetraethoxypropane (Sigma-Aldrich T9889; St. Louis) was employed as standard, whereas for TEAC 6-hydroxy-2,5,7,8-tetramethylchromane-2-carboxylic acid (Trolox; Aldrich Chemical Co 23881–3) was used. In both cases a standard curve was built and linear regression calculated. Other measurements used standards provided by the producer of the kit. Control serum was purchased from Controllab (Rio de Janeiro – Brazil). Standards and control samples were assayed in every batch to ensure laboratory testing reliability. All commercial kits and reagents were approved by Brazilian Regulatory Agency (ANVISA).

### Body composition assessment

Body composition was assessed by measuring body weight and height before and after the experiment. Body fat percentage was estimated from measurements of triceps, abdominal and suprailiac skin folds. A Lange® caliper was used to measure subcutaneous tissue, and the fractionation of body weight (body fat percentage and lean mass) was determined according to the equation proposed by Guedes [24]. The upper muscle area (UMA) was also calculated by measuring right arm diameter and triceps skin fold [25]. In order to confirm reliability, such tests were performed in duplicates and the correlations found were 0.88 and 0.94.

### Muscular strength and endurance assessment

A standard isotonic bench press (Physicus; Auriflama; São Paulo- Brazil) was used for the isotonic bench press tests: One-repetition maximum and muscle endurance tests. Specifically, the muscular endurance test consisted of executing the bench press at 80% 1RM until reaching maximum volunteer fatigue, and then the replicates obtained were multiplied by the shifted load in Kg [26]. In order to confirm reliability, such tests were performed in duplicates and the correlations found were 0.90 and 0.96.

### Statistical analysis

All data are presented as means and standard deviations. Sample size was pre-calculated in order to ensure statistical power (0.80) to be a minimum of 7 subjects per group. The statistical analysis was initially done by the Shapiro-Wilks normality test (W test) to verify if the sample showed normal distribution. Differences between groups were analyzed using Friedman test and Dunn post-test to compare age, upper muscle area, body composition, muscular strength and endurance, whist comparison for TBARS, TAS, CPK, uric acid, creatinine, and urea were performed using ANOVA with Tukey post-hoc test. Intra group (post x pre) analyzes were performed by paired t-Student test. In all calculations, a critical level of *p* < 0.05 was fixed. GraphPad Prism® software was used for the analysis.

## Results

### Body composition

There were no significant changes in weight, body fat, or lean body mass from baseline to post-supplementation values in the GC, GP or COT. Values for these parameters are displayed in Table 2.

**Table 2:** Anthropometric data before and after creatine supplementation and resistance training

**Group**	**Height (cm)**		**Weight (kg)**		**Body fat (%)**		**Lean Body Mass (kg)**	
	**Pre**	**Post**	**Pre**	**Post**	**Pre**	**Post**	**Pre**	**Post**
**GC**	182 ± 6	182 ± 6	79 ± 10	80 ± 8	16.5 ± 6.2	16.2 ± 5.5	66 ± 5	67 ± 23
**GP**	181 ± 5.4	181 ± 5.4	80 ± 11	78 ± 9	12.3 ± 6.1	11.1 ± 5.9	69 ± 9	69 ± 9
**COT**	178 ± 6.9	178 ± 6.9	73 ± 13	75 ± 13	14.1 ± 7.7	13.8 ± 9.3	62 ± 6	64 ± 5

Values are expressed as mean ± SD; GC= creatine supplemented athletes; GP= placebo (malthodextrin) supplemented athletes; COT= non-supplemented control athletes.

### UMA and muscular tests

There was no significant change in UMA from baseline to post measurement in the GC, GP or COT. However, there was significant increase in muscular strength (bench press) for GC (54 ± 9 kg and 63 ± 10 kg, respectively; p = 0.0356), but not for GP (54 ± 19 kg and 58 ± 17 kg, respectively) or COT (48 ± 12 kg and 56 ± 11 kg, respectively). No significant differences in muscular endurance (bench press) were found, as seen in Table 3.

**Table 3:** Muscular area (UMA), strength, and muscle endurance before and after creatine supplementation and resistance training

**Group**	**UMA (cm**^**2**^**)**		**Strength (kg)**		**Muscle endurance (kg)**	
	**Pre**	**Post**	**Pre**	**Post**	**Pre**	**Post**
**GC**	53 ± 9	58 ± 5	54 ± 9	63 ± 10^**a**^	320 ± 215	368 ± 186
**GP**	56 ± 11	60 ± 12	54 ± 19	58 ± 17	311 ± 142	272 ± 83
**COT**	49 ± 8	52 ± 7	48 ± 12	56 ± 11	306 ± 148	279 ± 130

Values are expressed as mean ± SD; GC= creatine supplemented athletes; GP= placebo (malthodextrin) supplemented athletes; COT= non-supplemented control athletes. ^**a**^ P value = 0.0356 x Pre.

### Creatine phosphokinase (CPK), creatinine and urea

There were no post-training differences among groups for CPK, creatinine or urea. Likewise, no differences were seen in each group when comparing pre- and post-supplementation values for CPK, creatinine, or urea. Table 4 presents CPK, creatinine and urea values.

**Table 4:** Creatine Phosphokinase (CPK), Creatinine and urea levels before and after creatine supplementation and resistance training

**Group**	**CPK (U/l)**		**Creatinine (mg/dl)**		**Urea (mg/dl)**	
	**Pre**	**Post**	**Pre**	**Post**	**Pre**	**Post**
**GC**	95 ± 32	153 ± 99	1.41 ± 0.77	1.47 ± 0.28	25 ± 6	38 ± 9
**GP**	111 ± 62	95 ± 49	1.03 ± 0.57	1.25 ± 0.23	26 ± 9	38 ± 11
**COT**	129 ± 71	121 ± 78	1.10 ± 0.88	1.27 ± 0.23	24 ± 5	35 ± 9

Values are expressed as mean ± SD; GC= creatine supplemented athletes; GP= placebo (malthodextrin) supplemented athletes; COT= non-supplemented control athletes.

### Oxidative stress makers

Table 5 summarizes levels of oxidative stress markers. A significant 61% increase on the post-training mean value of uric acid was found for GC, when compared to GP and COT (7.4 ±1.6 mg/dL, 6.7 ± 2.3 mg/dL and 6.7 ± 1.2 mg/dL, respectively; p = 0.025), whereas no differences were seen for TBARS. Nevertheless, TAS values were significantly reduced for GC, in comparison to GP or COT (0.60 ± 0.19 mmol/L, 0.75 ± 0.22 mmol/L and 0.87 ± 0.42 mmol/L, respectively; p = 0.001). Furthermore, GC showed a significant 46% decrease for TAS, when comparing pre- and post-supplementation time (1.11 ± 0.34 mmol/L for pre- vs. 0.60 ± 0.19 mmol/L for post-supplementation time; p=0.025).

**Table 5:** Effect of creatine supplementation and resistance training on oxidative stress markers

**Group**	**Uric Acid (mg/dl)**		**TBARS (ng/dl)**		**TAS (mmol/l)**	
	**Pre**	**Post**	**Pre**	**Post**	**Pre**	**Post**
**GC**	4.6 ± 1.0	7.4 ± 1.6^**a**^	216 ± 79	271 ± 92	1.11 ± 0.34	0.60 ± 0.19^**b**^
**GP**	4.4 ± 1.1	6.7 ± 2.3	209 ± 104	255 ± 77	0.91 ± 0.28	0.75 ± 0.22
**COT**	5.1 ± 0.9	6.7 ± 1.2	211 ± 96	264 ± 109	0.89 ± 0.15	0.87 ± 0.42

Values are expressed as mean ± SD; GC= creatine supplemented athletes; GP= placebo (malthodextrin) supplemented athletes; COT= non-supplemented control athletes; TBARS= Thiobarbituric Acid Reactive Substances; TAS= Total Antioxidant Status; ^**a**^*P* value = 0.025 vs. Pre; ^**b**^*P* value = 0.001 vs. Pre.

Additionally, the differences between post- and pre-supplementation values were calculated and revealed that GC group displayed significant higher levels than GP and COT of uric acid (2.77 ±1.70 mg/dL, 2.26 ± 2.38 mg/dL and 1.00 ± 1.03 mg/dL, respectively; p = 0.0276) and strength (8.30 ± 2.26 kg, 5.29 ± 3.77 kg, and 5.29 ± 2.36 kg, respectively; p = 0,0209), and lower levels of TAS (−0.51 ± 0.36 mmol/L, -0.11 ± 0.37 mmol/L and −0.02 ± 0.50 mmol/L, respectively; p = 0.0268). On the other hand, no differences were found for TBARS (Table 6).

**Table 6:** Differences (post- vs. pre-training) on oxidative stress markers and strength

**Group**	**Uric Acid (mg/dl)**	**TBARS (ng/dl)**	**TAS (mmol/l)**	**Strength (kg)**
**GC**	2.77 ± 1.70^**a**^	55 ± 98	−0.51 ± 0.36^**b,c**^	8.30 ± 2,26^**d,e**^
**GP**	2.26 ± 2.38	40 ± 118	−0.11 ± 0.37	5.29 ± 3.77
**COT**	1.00 ± 1.03	48 ± 130	−0.02 ± 0.50	5.29 ± 2.36

Values are expressed as mean ± SD; GC= creatine supplemented athletes; GP= placebo (malthodextrin) supplemented athletes; COT= non-supplemented control athletes; TBARS= Thiobarbituric Acid Reactive Substances; TAS= Total Antioxidant Status; ^**a**^*P* value = 0.0276 vs. COT; ^**b**^*P* value = 0.0268 vs. COT; ^**c**^*P* value = 0.0253 vs. GP; ^**d**^*P* value = 0.0209 vs. COT; ^**e**^*P* value = 0.0283 vs. GP.

## Discussion

The present study highlights a significant increase in the rate of maximum force production achieved by the Cr-supplemented group, confirming the ergogenic effect of Cr supplementation previously described [27-29]. However, no significant differences in body weight, lean body mass and arm muscle area were observed in the GC group after Cr supplementation and resistance training. These data suggest a specific effect of Cr supplementation associated with the type of periodization used. Creatine acts in the energy production process; on that account, increase in strength observed in the GC group was most probably the result of improved ATP resynthesis efficiency leading to increased intramuscular ATP concentration [30], and not from muscle hypertrophy. These data suggest the applicability of Cr supplementation combined with resistance training in athletes of specific modalities (boxing, martial arts, tennis, soccer, etc.) that require power growth without increase in body weight.

Follow-up and evaluation of the athletes was conducted by a sports medicine doctor before, during, and after intervention. No clinical alterations or muscle injuries were observed in any subject of any group. In fact, many studies suggest that Cr supplementation within the recommended dosage regimens is not associated with any negative effects to healthy subjects [2,17,31,32].

However, in the last decade Cr supplementation has been surrounded by myths linked to several health disorders, particularly renal function. These concerns are related to plasma creatinine concentrations [33]. In the present study, mean plasma creatinine levels increased upon completion of the supplementation period; though not significantly, suggesting that renal function in these individuals remained satisfactory.

The safety of Cr supplementation has been demonstrated in a number of studies over the years. For example, in a study with 20 men aged between 19 and 28 years (ingesting 20 g/day Cr for 5 days), Arnold et al. [34] observed that increased muscle glycogen was related to intracellular Cr levels, yet no side effects were detected.

The present study aimed at verifying the effects of Cr supplementation over oxidative stress markers in healthy young male athletes. TBARS, a lipid peroxidation marker - and therefore oxidative stress - was assayed, as well as total antioxidant capacity, a method that measures the consumable antioxidant defenses of subjects. Moreover, considering that resistive exercise may impose situations of physiological ischemia to body tissues, followed by oxygen upload, ischemia-reperfusion syndrome (SIR) might occur and become an additional source of free radicals, so uric acid was assessed, since it is a byproduct of SIR.

Conversely, TBARS levels were within normal limits for the three groups, which did not differ from each other. These data suggest no involvement of oxidative stress, but one must bear in mind that a possible lack of specificity in this method and technical artifacts may overcame the differences among groups.

Likewise, an increase in uric acid in all groups after the periodization protocol was observed, which was only statistically significant in the GC group. This fact has been widely described in a number of studies showing that plasma uric acid levels rise in ischemia-reperfusion events. The elevation in uric acid concentration suggests the occurrence of ischemia-reperfusion syndrome induced by resistance training and the consequent free radical production. Actually, McBride et al. [13] suggest that muscle contraction caused by excessive resistance exercise may result in ischemia-reperfusion in active muscles. Moreover, high-intensity physical activity was observed to promote ATP degradation, with consequent plasma hypoxanthine and uric acid increase.

However, TAS values suggested a significant reduction in antioxidant defense in the GC group compared to the other groups. In this sense, significant strength gains in group GC may have promoted an increase in the energy production mechanism owing to the large capacity for ATP resynthesis in cells under Cr supplementation. This situation may be favorable for the manifestation of ischemia-reperfusion syndrome, with increased uric acid and hydroxyl radical production causing the mobilization of antioxidant reserves - thereby reducing TAS - to prevent oxidative stress.

These results conflict with those presented by Guézennec et al. [35], who suggested that Cr supplementation results in decreased hypoxanthine and urate production, as indicated by the reduction of ammonia concentration and increased performance. In this respect, these authors concluded that Cr supplementation had a sparing effect on purines. Likewise, Souza Júnior and Pereira [36] suggested that Cr may act as an energy buffer, either indirectly via increased intracellular phosphocreatine concentration, which may lessen formation of ATP degradation products, or because of the direct effects of arginine found in its molecular structure. However, we believe that even if Cr plays a role preventing ATP depletion, the energy production required for intense muscle activity will always be maximal and thus exacerbate purine degradation, since increasing the capacity for ATP resynthesis through Cr supplementation would make more ATP available for degradation.

We believe that Cr supplementation boosts energy production and consequently increases hypoxanthine formation, resulting in free radical production, which in turn promotes consumption of antioxidant reserves.

## Conclusion

We conclude that Cr supplementation associated to a specific resistance program promotes a significant increase in muscular strength without changes in body composition. However, the significant increase in uric acid and the decrease in TAS displayed for GC subjects, suggest that Cr supplementation promotes free radical generation, and therefore, an over consumption of antioxidant reserves in order to defend themselves.

These results suggest that creatine supplementation, despite promoting acute muscle strength improvement, may be harmful as it induces oxidative stress and decreases total antioxidant status. Nevertheless, further research is needed in this field to fully attest these results.

## Abbreviations

1-RM: 1-repetition maximum; CPK: Creatine phosphokinase; COT: Group of control athletes with no supplementation or placebo; Cr: Creatine; GC: Group of athletes supplemented with creatine; GP: Group o athletes who were given placebo; H_2_O_2_: Hydrogen peroxide; O_2_^·^: Superoxide radicals; OH·: Hydroxyl radicals; TAS: Total antioxidant status; TBARS: Thiobarbituric acid reactive species; UMA: Upper muscle area.

## Competing interests

All authors declare that they have no competing interests.

## Authors’ contributions

MC and SP have idealized the study and are responsible for the final form of the manuscript; SPTD, DMC, MC and LMT conducted the exercise training, supplement administration, sample collection and the draft of the manuscript; JLFV, SP, FV, and EDA performed laboratory testing, statistical analysis, and contributed to the draft of the manuscript. All authors read and approved the final manuscript.
